# A Nurse Case Management HIV Prevention Intervention (Come As You Are) for Youth Experiencing Homelessness: Protocol for a Randomized Wait-list Controlled Trial

**DOI:** 10.2196/26716

**Published:** 2021-05-21

**Authors:** Diane Santa Maria, Marguerita Lightfoot, Adey Nyamathi, Michael Businelle, Mary Paul, Yasmeen Quadri, Nikhil Padhye, Jennifer Jones, Margarita Calvo Armijo

**Affiliations:** 1 Cizik School of Nursing University of Texas Health Science Center at Houston Houston, TX United States; 2 Center for AIDS Prevention Studies and UCSF Prevention Research Center University of California San Francisco San Francisco, CA United States; 3 Sue & Bill Gross School of Nursing University of California Irvine Irvine, CA United States; 4 TSET Health Promotion Research Center University of Oklahoma Health Sciences Center Oklahoma City, OK United States; 5 Baylor College of Medicine Houston, TX United States; 6 Department of Family and Community Medicine Baylor College of Medicine Houston, TX United States

**Keywords:** HIV prevention, nurse case management, motivational interviewing, homelessness, youth, just-in-time-adaptive intervention, ecological momentary assessment

## Abstract

**Background:**

Youth experiencing homelessness are more likely than housed youth to experience premature death, suicide, drug overdose, pregnancy, substance use, and mental illness. Yet while youth experiencing homelessness are 6 to 12 times more likely to become infected with HIV than housed youth, with HIV prevalence as high as 16%, many do not access the prevention services they need. Despite adversities, youth experiencing homelessness are interested in health promotion programs, can be recruited and retained in interventions and research studies, and demonstrate improved outcomes when programs are tailored and relevant to them.

**Objective:**

The study aims to compare the efficacy of a nurse case management HIV prevention and care intervention, titled Come As You Are, with that of usual care among youth experiencing homelessness aged 16 to 25 years.

**Methods:**

The study is designed as a 2-armed randomized wait-list controlled trial. Participants (n=450) will be recruited and followed up for 9 months after the intervention for a total study period of 12 months. Come As You Are combines nurse case management with a smartphone-based daily ecological momentary assessment to develop participant-driven HIV prevention behavioral goals that can be monitored in real-time. Youth in the city of Houston, Texas will be recruited from drop-in centers, shelters, street outreach programs, youth-serving organizations, and clinics.

**Results:**

Institutional review board approval (Committee for the Protection of Human Subjects, University of Texas Health Science Center at Houston) was obtained in November 2018. The first participant was enrolled in November 2019. Data collection is ongoing. To date, 123 participants have consented to participate in the study, 89 have been enrolled, and 15 have completed their final follow-up.

**Conclusions:**

There is a paucity of HIV prevention research regarding youth experiencing homelessness. Novel and scalable interventions that address the full continuum of behavioral and biomedical HIV prevention are needed. This study will determine whether a personalized and mobile HIV prevention approach can reduce HIV risk among a hard-to-reach, transient population of youth at high risk.

**International Registered Report Identifier (IRRID):**

DERR1-10.2196/26716

## Introduction

### HIV Risks Among Youth Experiencing Homelessness

A number of systemic barriers and risk behaviors drive high HIV infection rates among youth experiencing homelessness. Youth experiencing homelessness have earlier sexual debuts; are more likely to have multiple sexual partners; and trade sex for food, shelter, money, drugs, or alcohol [1,2]. They are more likely to use substances before sex, are less likely to use condoms, and are overrepresented by youth who identify as men who have sex with men; each of these characteristics increase risk for HIV [3,4], and those who trade sex are at high risk for HIV infection as they are rarely able to negotiate condom use due to the power dynamics [5] and often lack knowledge about biomedical advances in HIV prevention such as preexposure prophylaxis and nonoccupational postexposure prophylaxis [6]. In a recent 7-city (Houston, Denver, St. Louis, Phoenix, Los Angeles, San Jose, New York City) study of 1427 youth experiencing homelessness (58% male, 81% youth of color, 31% lesbian, gay, bisexual, transgender, queer [LGBTQ]), 71% of participants had little to no knowledge of preexposure prophylaxis [7]. Reassuringly, 53% of study participants had undergone HIV testing in the preceding 3 months [7]. Unfortunately, youth experiencing homelessness experience sexual assault and forced sex at high rates (22% and 24% respectively); yet only 29% received a postsexual assault examination which is when they could have received nonoccupational postexposure prophylaxis and sexually transmitted infection (STI) treatment [8]. The Society for Adolescent Health and Medicine has recommended the development of screening tools, skill-building interventions, and accessible preexposure prophylaxis delivery models for all youth and young adults, particularly those experiencing disparities [9].

### Implications of Mental Health and Substance Use on HIV Risk

HIV risk among youth experiencing homelessness is further exacerbated by multiple comorbid conditions, including mental illness and substance use. Suicide is one of the leading causes of death among youth experiencing homelessness [10], with suicide attempt rates ranging from 12% to 48% [11-13]. Rates of depression and posttraumatic stress among youth experiencing homelessness vary across studies with ranges from 8% [14] to 61% [15] and 5% to 48% [16-18], respectively. A recent study [19] found that 42% of youth experiencing homelessness reported being moderately to severely stressed, 48% experienced mental distress, 48% had depression, and 23% had posttraumatic stress. Depression among youth experiencing homelessness may be due to a lifetime of adversity, abuse, neglect, and housing instability [20,21]—all of which can lead to inhibition and riskier sexual decision making and behavior [22]. Furthermore, rates of substance use are twice those of housed youth [3,23]. In one study [24], 86% of youth experiencing homelessness (n=173) met the Diagnostic and Statistical Manual of Mental Disorders, fourth edition, criteria for a substance use disorder compared with only 14.2% in the general young adult population [25]; drug overdose is a leading cause of death among youth experiencing homelessness [10]. HIV prevention efforts should address mental health and the intersection of substance use and sexual behaviors.

### Barriers to Health Care Access

Youth experiencing homelessness are underserved by the health care system for several reasons. Structural barriers include transportation, lack of health insurance, and costs [26]. Youth also suggest that fear of or past experiences of being judged, dismissed, or discriminated against by health care professionals reduce utilization [27]. Other barriers to health care access include fear of social service agency notification or legal intervention, lack of familiarity with health care resources, and lack of affordability [28]. As a result, youth experiencing homelessness often interact with the health care system at lower rates than their housed peers and frequently overutilize emergency departments for care while experiencing reduced access to prevention services [3]. Therefore, it is essential to increase access to and availability of HIV prevention services, and these services should be colocated with other service programs to foster trust and increase accessibility for youth experiencing homelessness [29-31]

### Interventions for Youth Experiencing Homelessness

A recent systematic review [32] of interventions to prevent HIV among youth experiencing homelessness highlighted the paucity of HIV prevention research and concluded that more research is necessary. Interventions for youth experiencing homelessness should include the full continuum of behavioral and biomedical HIV prevention, including HIV and STI screening and treatment, preexposure prophylaxis, and nonoccupational postexposure prophylaxis [33]. Engagement in these prevention services requires interventions to increase preexposure prophylaxis awareness, screen for preexposure prophylaxis eligibility, promote condom use, provide assistance with health care navigation that includes care for mental health and substance use issues [34], and address transportation and health insurance challenges. Individuals impacted by mental illness, homelessness, and substance use have greater preexposure prophylaxis uptake and adherence when these abovementioned issues are also addressed [35]. Nurse case management is an evidence-based strategy that has been effective in addressing the multifaceted and complex health and social challenges of HIV prevention among youth experiencing homelessness [27,36-38]

### Nurse Case Management

A nurse-led intervention allows for multiple HIV prevention services to be delivered during a single visit (eg, preexposure prophylaxis, nonoccupational postexposure prophylaxis, lab draws, STI testing and treatment), which may increase adherence. This is particularly important as being homeless can decrease effectiveness of linkages to care [39] as opposed to providing that care at the point of contact. Nurse case management has been efficacious in reducing drug use among methadone users [40] and youth experiencing homelessness [41], improving hepatitis B vaccination rates [42], and facilitating HIV care coordination [43]. This comprehensive approach of simultaneously addressing concomitant problems (eg, mental health, substance use, and housing needs), incorporating the full continuum of behavioral and biomedical HIV prevention, is a promising strategy for engaging youth experiencing homelessness in HIV prevention. Given the widespread integration of nurses into current HIV programs serving youth experiencing homelessness, nurse-led interventions are likely scalable and can be integrated into existing HIV prevention programs.

To further engage youth experiencing homelessness, they should be met “where they are” [35] and interventions should be implemented in collaboration with existing health and social service providers by colocating the study activities at drop-in centers, shelters, and service providers that are highly used by youth experiencing homelessness. This strategy is particularly important given that past-month use of a drop-in center has been shown to predict HIV and STI testing [44], increase service utilization, and improve HIV-related outcomes [31]. These findings support the potential of delivering HIV prevention in drop-in centers and shelters to connect youth experiencing homelessness to other underutilizing services and health care. Moreover, drop-in centers may be a preferred HIV prevention service location of youth experiencing homelessness [31]. By integrating the delivery of HIV risk reduction interventions into already-established social services, we may enhance HIV prevention, increase retention, and improve access to mental health, substance use, and housing services.

### Motivational Interviewing and Behavior Feedback

Nurse-led interventions can also integrate evidence-based strategies that have proven effective in increasing motivations for behavior change. Increasing motivation is particularly important for populations that are potentially overwhelmed by multifaceted and complex health and social challenges. Motivational interviewing is a person-centered counseling style that aims to strengthen a person’s motivation and commitment to change and addresses ambivalence about behavior change [45]. Motivational interviewing has been successfully used with youth to improve uptake of and adherence to health behaviors resulting in reduced alcohol [46] and substance use [47], and increased condom [48] and contraceptive use [49]. Youth experiencing homelessness are self-reliant, can be challenging to engage, and may be distrustful of adults due to past trauma and victimization on the streets [50,51]. Motivational interviewing strategies can strengthen the relationship between the youth experiencing homelessness and providers to evoke participant driven HIV prevention goals [52].

Behavioral feedback technology might also increase motivation to change behavior as tailored and targeted feedback could further engage youth experiencing homelessness. Smartphone-based daily ecological momentary assessments have been used with youth experiencing homelessness. Instant feedback enhances cognitive appraisal of health-seeking and coping behaviors and increases motivation in youth experiencing homelessness [52,53]. Like many adolescents and young adults, youth experiencing homelessness underestimate their HIV risk [7], suggesting that self-monitoring may assist in aligning their behaviors with their perceived HIV risk. Immediate self-monitored behavioral feedback has been found to increase condom use [52,54]. A high number of youth have phones, and young adults, in general [55-57], and youth experiencing homelessness, specifically, have a preference to use technology [58]. A review [59] of 42 studies showed high ecological momentary assessments completion rates (78%) among youth. Prior studies [60] have found similar high adherence rates (82%-87%) with homeless and vulnerable populations.

### Objectives

This study describes the design and implementation of a nurse case management intervention (Come As You Are) efficacy trial with youth experiencing homelessness aged 16 to 25 years who received the active intervention or usual care. The intervention aims to increase uptake of HIV prevention strategies (eg, pre- and postexposure prophylaxis uptake, HIV testing, STI screening and treatment, sober sex, and condom use) when compared with usual care youth experiencing homelessness immediately postintervention and 3, 6, and 9 months postintervention. The study also aims to determine whether the intervention improves mental health symptoms, substance use, and housing status. Additionally, we will assess whether health seeking, coping, HIV risk perception, pre- and postexposure prophylaxis barriers and facilitators, and condom self-efficacy mediate the effect of the intervention on uptake prophylaxis, condom use, and HIV/STI testing. This protocol paper describes the study design, intervention, recruitment, and retention strategies.

## Methods

### Study Design

**Figure 1:**
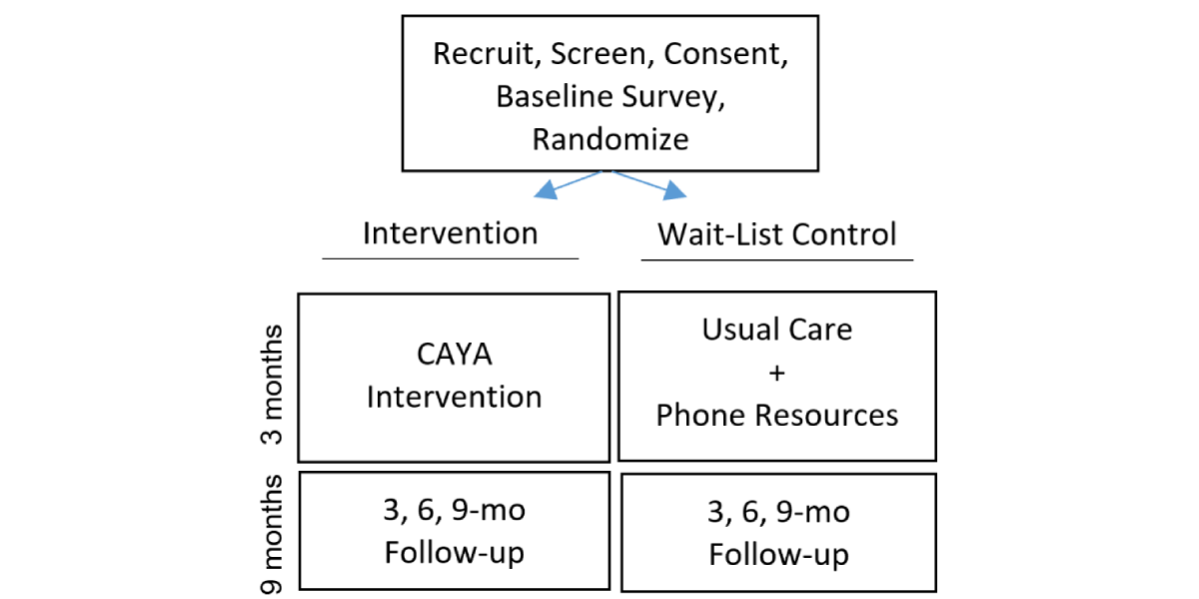
Study flow diagram. CAYA: Come As You Are.

This study uses a 2-armed randomized controlled trial design with a wait-list control group to determine the efficacy of the intervention compared to usual care. The primary outcomes are the uptake of HIV prevention strategies (preexposure prophylaxis and nonoccupational postexposure prophylaxis, HIV and STI testing, and condom use). Secondary outcomes of the intervention include the impact on mental health, substance use, and housing status. Follow-up surveys are conducted immediately after the 3-month intervention period and 3, 6, and 9 months postintervention (Figure 1).

Participants are randomly assigned to the intervention or wait-list control arm using a computer-generated blocked 2:1 allocation. By the end of the recruitment period, we anticipate that 300 participants will be randomized to the intervention arm and 150 randomized to the wait-list control arm. Participants are informed in which group they are allocated after completing the baseline survey.

### Recruitment

Utilizing numerous recruitment sites in Houston—drop-in centers, shelters, local youth experiencing homelessness service locations, clinics, federally qualified health care centers in locations with a high concentration of homelessness, magnet (eg, hot meal) events, mobile clinics, and street outreach—will increase generalizability of the findings by including a sample of both connected and disconnected youth experiencing homelessness. These recruitment sites serve young men, women, families, and LGBTQ youth. We make use of group-based study introduction sessions, flyers, and recruitment letters at the agencies, clinics, street outreach, and the website and Facebook pages of the agencies and Homeless Youth Network of Houston. We have used these methods successfully in previous studies [61]. The research staff will maintain a consistent, weekly presence at the recruitment sites throughout the study to facilitate both recruitment and follow-up efforts. In response to COVID-19 physical distancing requirements and shelter closures, we are also using snowballing participant referral techniques and online advertisements.

### Inclusion and Exclusion Criteria

Our sample is limited to youth, 16 to 25 years old, to align with youth homelessness services providers and adolescent risk behavior studies [7], current guidelines for preexposure prophylaxis and nonoccupational postexposure prophylaxis use [62,63], and evidence that experiencing homelessness as a young adult under 26 years of age is associated with heightened sexual risk behaviors and substance use [33]. Individuals are eligible to be included if they (1) are 16 to 25 years old, (2) speak English, (3) are experiencing homelessness, and (4) are not planning to move out of the Houston metropolitan area during the study.

Experiencing homelessness is defined as having slept on the streets, in a place not meant for habitation, in a shelter, hotel or motel, or with someone where they cannot stay for more than 30 days (eg, couch surfing). Youth experiencing homelessness may stay in emergency shelters or on the streets (eg, parks and tent cities); in abandoned or vacant buildings or apartments; temporarily with friends, family, or acquaintances; or in rented hotel or motel rooms [35], and they can go to great lengths to stay hidden from the dangers of victimization [50]. This broad definition of homelessness aligns with the McKinney-Vento Homeless Assistance Act of 1987 [64], which allows us to account for the transiency and instability of housing experienced by youth experiencing homelessness and increases the generalizability of the study findings.

Youth with very low literacy (Rapid Estimate of Adult Literacy in Medicine-Short Form [65] health literacy assessment score <4)[1] are excluded from the study due to the need to independently read the daily smartphone-based assessments. Additionally, youth who are noticeably intoxicated or experiencing acute mental distress are encouraged to be screened for enrollment at a later time to assure safety and acute needs are met prior to enrollment. Youth are connected to services at the recruitment sites for acute needs.

### Study Enrollment

Due to the transient nature of the study population, study participants are being enrolled in a stepwise process that takes place over the course of 3 weeks. The first step entails a thorough review of the consent form and collection of contact information including a photo, and the second step consists of baseline data collection. During the third step, participants receive the study phone and are notified of study group assignment. Intervention participants receive the first intervention session on the same day that they receive the study phone. During the COVID-19 pandemic, consenting and the baseline survey are being completed remotely as needed to reduce the face-to-face study visits to only 1 enrollment visit.

### Intervention Description

**Figure 2:**
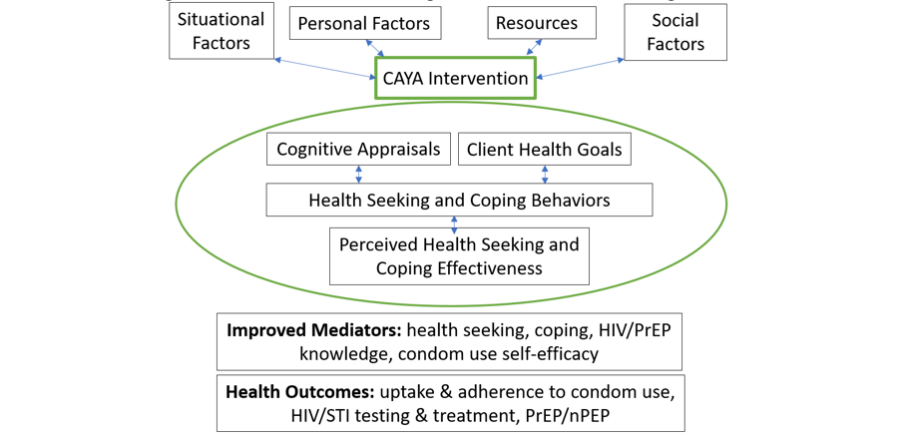
Motivational interviewing enhanced case management. CAYA: Come As You Are; HIV: human immunodeficiency virus; PrEP: preexposure prophylaxis; nPEP: nonoccupational postexposure prophylaxis; STI: sexually transmitted infection.

The *Come As You Are* intervention is based on the Comprehensive Health Seeking and Coping Framework (CHSCF; Figure 2), which describes how the nurse and client work together to mutually develop goals and strategies to improve health in a context of nonjudgmental acceptance. Accomplishment of goals occur by addressing cognitive appraisals (clarifying misconceptions), promoting health seeking, and addressing knowledge and coping behaviors that incorporate the situational, personal, social, and resource needs affecting health. The intervention involves coordinated, individualized, comprehensive care delivered by a nurse that includes a comprehensive health assessment, mutual care plan development, prevention education, and health and social service navigation [36-38,41]. An individualized, rather than group based, intervention was chosen for this study due to the heterogeneity of youth experiencing homelessness and their risk behaviors and the challenges associated with group session designs, including low attendance [66]. The intervention has 2 main components: 6 face-to-face sessions with a study nurse and a behavioral assessment and feedback app. Additionally, booster calls are made monthly for 3 months following the last face-to-face session. This intervention is guided by the CHSCF [67] and uses motivational interviewing [45,68] strategies to promote behavior change and uptake of HIV prevention strategies by facilitating goal setting and evoking change talk.

### Come As You Are Intervention Group

#### Face-to-Face Sessions

The 6 biweekly 1-hour face-to-face sessions are designed to meet the complex, individual, multilevel health and social needs of homeless youth to align with extant literature demonstrating the strong connection between HIV risk behaviors and mental health, substance use, and housing [69]. During each session, the nurse uses motivational interviewing strategies to assess current mental health, substance use, and housing needs to establish a plan of care to assist youth in generating HIV prevention behavioral goals while addressing barriers. During these sessions, the nurse conducts an HIV risk profile and preexposure prophylaxis eligibility assessment and guides discussions about goal attainment strategies (Table 1). Six sessions allow for the development of the nurse-client relationship and for adequate time to establish, monitor, and maintain HIV prevention goals [70]. The motivational interviewing strategies allow the sessions to be youth-driven and tailored to the individual’s needs based on their current HIV risk behaviors, HIV status, behavioral goals, and motivation level. In response to the challenges experienced during the COVID-19 pandemic, the nurses also offered to conduct these individual sessions via videochat or phone call as needed (when shelter-in-place orders are enacted or service sites are closed) to protect their clients.

**Table 1:** Come As You Are session description.

Number	Session title	CHSCF^a^ constructs	Discussion topics	Session goals
1	Introduction and needs assessment	Situational, personal, social factors, and resources	Review HIV^b^ risk behavior and prevention strategy (condoms, HIV/STI^c^ testing, treatment, preexposure prophylaxis, nonoccupational postexposure prophylaxis) knowledge, attitudes, beliefs, and self-efficacy	Establish rapport; assess HIV risk, preexposure prophylaxis eligibility
2	HIV prevention strategies and goal setting	Nursing goals	Review personal HIV risk; use motivational interviewing to discuss risk reduction and prevention strategies; evoke change talk	Select HIV prevention goals and action plan
3	Behavioral feedback and goal alignment	Health seeking and coping behaviors	Identify gaps between goals and behaviors (eg, self-management, coping, health care engagement); evoke change talk	Revise/reinforce plan to meet/maintain HIV prevention goals
4	Addressing facilitators and barriers	Perceived behavior adherence and coping effectiveness	Review goals and action plan; use motivational interviewing to discuss personal HIV prevention behavior change facilitators and barriers; evoke change talk	Revise/reinforce HIV prevention goals and action plan to increase facilitators
5	Establishing a medical home	Immediate health outcomes	Review goals and action plan; discuss local clinic preferences and schedule well-check as indicated; evoke change talk and behavioral maintenance	Revise/reinforce HIV prevention goals, action plan, follow-up care plan
6	Moving toward health and wellbeing	Long-term health outcomes	Review goals and action plan; identify additional health, housing, work, and education needs and goals; evoke change talk and behavioral maintenance plans	Reinforce HIV prevention goals and action plan

^a^CHCSF: Comprehensive Health Seeking and Coping Framework.

^b^HIV: human immunodeficiency virus.

^c^STI: sexually transmitted infections.

#### HIV Risk Profile and Preexposure Prophylaxis Eligibility Assessment

HIV status is assessed at baseline using a rapid, finger stick HIV test. During the first intervention session and at the beginning of each subsequent session, the nurse uses a screening tool developed from the Centers for Disease Control and Prevention 2014 Clinical Practice Guidelines to assess preexposure prophylaxis eligibility. This screener is used to assess preexposure prophylaxis eligibility based on the participants’ HIV risk behaviors (eg, having an HIV-positive sexual partner, a recent bacterial STI, number of sex partners, a history of inconsistent or no condom use, injection drug use, or trade sex) to determine whether recent behaviors warrant preexposure prophylaxis as a possible intervention for HIV prevention. preexposure prophylaxis and HIV clinical care guidelines and standing orders under the supervision of a health care provider are incorporated into the 6 nurse sessions. The care plan for these participants includes coordinating access to, uptake of, and adherence to preexposure prophylaxis, and promoting HIV-preventing behaviors (eg, HIV/STI testing, using condoms, reducing sexual partners, reducing intravenous drug use, engaging in sober sex, and avoiding trade sex). When a youth is HIV-negative and preexposure prophylaxis–eligible, the nurse discusses what preexposure prophylaxis is, how it works, its risks and benefits, and the implications of its use (eg, follow-up lab work and appointment schedule) to promote shared decision making. When participants are interested in receiving preexposure prophylaxis, the nurse works with the preexposure prophylaxis navigator who accompanies the youth to the preexposure prophylaxis appointment to begin lab work and assists with completing an application form to cover preexposure prophylaxis medication costs.

#### HIV Prevention Goal Setting

The nurse uses motivational interviewing strategies to evoke youth-driven HIV prevention goals and behavior change talk. Each session includes personalized HIV prevention education (ie, personal risk behaviors and prevention strategies) and goal setting. Session appraisals help align the youth’s goals with their current behaviors and evoke personal motivation to adopt and maintain HIV prevention strategies. For youth who are not preexposure prophylaxis–eligible, the nurse promotes the adoption or maintenance of other HIV prevention strategies (ie, condom use).

#### Behavioral Interface

**Figure 3:**
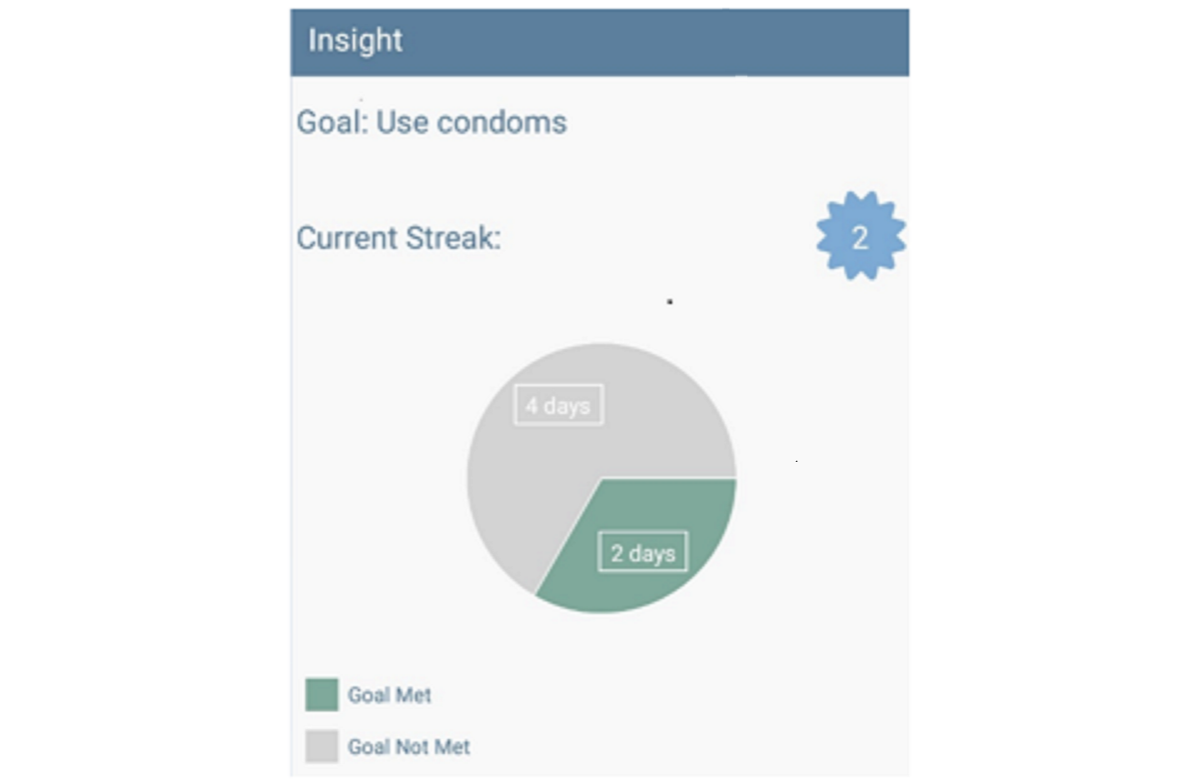
Example behavioral interface.

Building on the goal setting, participants in the intervention group complete a brief, daily ecological momentary assessment on their study-issued phone during the 3-month intervention delivery period. The ecological momentary assessment asks about sexual risk behaviors, sexual urges, stress, affect, social interactions, coping, and circumstances from the prior day (eg, where did you stay last night, sexual activity, substance use), and it takes less than 5 minutes to complete. Once the daily ecological momentary assessment is completed, the data populate a behavioral goal interface accessible by password on the study-issued smartphone (Figure 3). This interface provides a visual display based on the participant’s HIV prevention goals, and their behaviors as reported in the daily ecological momentary assessment. It allows the nurse and youth to review how current behaviors align with the health goals established during the Come As You Are session and facilitates discussion about the barriers and facilitators that impeded or enhanced uptake and adherence to HIV prevention strategies.

#### Booster Calls

After the face-to-face intervention sessions are completed, the team makes monthly booster calls to intervention participants on the study-issued phone for 3 months following the end of the individual sessions. During these calls, the team members inquire about the uptake and adherence to the HIV prevention strategies outlined during the Come As You Are sessions, asks if there are any other needs that they can address for the participant at that time, and helps the participant access and navigate services (ie, shelters, mental health counseling, health care) as needed.

### Wait-list Control Group

Youth in the control condition receive usual care from the recruitment sites including assistance with housing, food and clothing needs; basic health assessments and health care; limited anticipatory guidance; access to mental health counseling; substance use treatment referrals; and preexposure prophylaxis or nonoccupational postexposure prophylaxis referrals. Youth receiving usual care also receive a study phone and complete the baseline, 3-, 6-, and 9-month follow-up surveys. After the 12-month study period is completed, youth in the control group are invited to access the full Come As You Are intervention.

A Community Advisory Group and Youth Working Group provided input on study procedures, protocol implementation and will be active in the interpretation and dissemination of the findings to the community. These groups assisted in the development of study procedures, survey items, and recruitment materials. Additionally, they oversaw the creation of a local homeless resource guide to be given to all participants at the time of enrollment and preprogrammed into the study-issued phones. This guide contains location and contact information for local shelters, meals, social, legal, and education services, and clinics. These resources are available in paper version and preloaded to the phones (ie, suicide hotline, shelter contacts) for all participants. The Community Advisory Group are current service providers for youth experiencing homelessness. The Youth Working Group members are youth with lived homelessness experiences between the ages of 18 to 25 years.

### Data Collection Procedures

Data collected include baseline and follow-up survey data, HIV and STI test data, and data from smartphone-based daily ecological momentary assessments. Assessments are collected at baseline, at the end of treatment (3 months postbaseline), and 3, 6, and 9 months postintervention using REDCap (Vanderbilt University). The surveys are done in person or through a link to the survey sent to the participant via text message or email. The baseline survey assesses demographics (eg, age when first experiencing homelessness, duration experiencing homelessness, race and ethnicity, sexual orientation, gender identity), psychosocial factors, sexual behaviors, substance use, and mental health. The end of treatment assessment for the intervention group contains intervention process outcome items including what participants found to be the most and the least helpful, what made it easy or difficult to attend sessions. HIV and STI test data are collected at baseline, at the end of treatment, and 3, 6, and 9 months postintervention. Table 2 outlines measures used for primary outcomes. Youth with a positive STI test receives treatment and care coordination from the Healthcare for the Homeless Houston program, shelter clinics, or their medical home when preferred. Youth who test positive for HIV during the study will be linked to HIV care at a local clinic by the nurse.

**Table 2:** Outcome measures.

Construct		Scale or measure	Cronbach α
**Aim 1 outcomes**			
	Prophylaxis uptake	Preexposure prophylaxis uptake; nonoccupational postexposure prophylaxis uptake (NCM report, chart review)	—^a^
	Condom use	Youth Risk Behavior Survey [71] (condom use at last sexual encounter)	—
	HIV^b^/STI^c^ test uptake	Rapid HIV test; gonorrhea, chlamydia, syphilis tests	—
**Aim 2 outcomes**			
	Mental health	Kessler Psychological Distress Scale [72,73]	—
	Housing status	In a shelter, apt/house, with someone, outside, in a car	—
	Substance use	Texas Christian University Drug Screen II [74,75]	.89
**Exploratory outcomes^d^**			
	Health care engagement	Health care utilization, Medical Mistrust Index 2.1 [76]	—
	Condom-use self-efficacy	Condom Control beliefs [77]	.74-.83
	HIV risk perception	Perceived Risk of HIV scale [78]	.88
	Preexposure prophylaxis facilitators and barriers	Facilitators and Barriers to Preexposure Prophylaxis Use [79]	—
	Health seeking	General Help Seeking Questionnaire [80]	.83
	Coping	Derived from Ways of Coping Questionnaire [81]	—

^a^No data.

^b^HIV: human immunodeficiency virus.

^c^STI: sexually transmitted infections.

^d^Possible mediators based on Comprehensive Health Seeking and Coping Framework.

For HIV testing, we use the INSTI HIV-1/HIV-2 Rapid Antibody Test (99.5% sensitivity, 100% specificity; Biolytical Laboratories Inc)[82]. Urine specimens are collected and transported on the same day to a local clinic to test for *Chlamydia trachomatis* and gonorrhea (*Neisseria gonorrhoeae*), and results are shared with participants via phone call or in person. Blood samples are tested for syphilis (*Treponema pallidum*) using an antibody rapid immunochromatographic test (Syphilis Health Check, Trinity Biotech plc)[83]. Due to restrictions on in-person study visits during the COVID-19 pandemic, additional options for STI testing, including going to conveniently located partner clinics or receiving an at-home STI testing kits, are being provided to study participants as attrition mitigation strategies.

## Results

Institutional review board approval (Committee for the Protection of Human Subjects HSC-SN-18-0993) was obtained in November 2018. The first participant was enrolled in November 2019. Data collection is ongoing—to date, 130 participants have consented to the study, 110 have enrolled, and 15 have completed the final follow up—and expected to conclude in 2022.

## Discussion

This study will provide essential data on the efficacy of a 2-component nurse case management HIV prevention intervention (ie, nursing visits and smartphone based behavioral monitoring and feedback) among youth experiencing homelessness. Findings from the study will significantly contribute to the field of HIV prevention in a marginalized and hard-to-reach population. The intervention is designed to be scalable within the practical parameters of care currently provided through the Health care for the Homeless programs across the nation.

This project is innovative in several ways. It addresses the underutilized role of nurses, the most trusted professionals in the United States [84], in the HIV prevention team. Consequently, nurses’ abilities to provide HIV prevention services may reduce the need to refer youth experiencing homelessness to other health care providers, which can decrease referral no-show and treatment plan nonadherence. Combining nurse case management with motivational interviewing and behavioral feedback can potentiate motivation for adopting HIV prevention behaviors and address the full continuum of behavioral and biomedical HIV prevention with youth experiencing homelessness. If found to be effective, this intervention can be applied to improve existing youth experiencing homelessness HIV prevention program, maximizing the available resources and potential outcomes.

The eponymous intervention capitalizes on the “come as you are” approach endorsed in clinical guidelines [85] put forth by the National Healthcare for the Homeless Council and aligns with NIH and Ending the HIV Epidemic High Priority areas [86] for reducing HIV through behavioral prevention and access to services in high HIV prevalence and substance-using, high-risk populations. Additionally, the intervention facilitates coordination with youth experiencing homelessness service providers to meet mental health, substance use, and housing service needs and connects youth to the health care services, such as HIV and STI testing and treatment.

The potential benefits of study participation include increased knowledge about HIV transmission and increased uptake of prevention strategies. Participants may become more aware of how thoughts and feelings can affect one’s behaviors and improve uptake and adhere to HIV prevention goals. Research staff are provided with comprehensive lists of resources available to youth experiencing homelessness and receive extensive training on how to make referrals to appropriate resources if a participant indicates that they need services they are not otherwise receiving. Participants will have access to resources and contact information for services that will be preprogrammed into the study-issued smartphones provided to all participants throughout the duration of the study. Participants in the intervention arm may also benefit from linkages to care provided through the Come As You Are intervention. Through the HIV and STI testing offered to all participants, youth may become aware of a positive result and receive necessary treatment and linkages to care that they may not have otherwise received. If efficacious, this scalable intervention has the potential to be disseminated to young people experiencing homelessness across the country without requiring significant investments in infrastructure, equipment, or staff resources. Some potential challenges related to whether youth have access to a phone are present, though studies suggest that smartphone use among youth experiencing homelessness is similar to that in the general population, ranging from 47% to 78% [87,88]. Given the implementation of this study during the COVID-19 pandemic, there are unique learning opportunities related to executing a randomized control trial focused on HIV prevention during a global pandemic with youth experiencing homelessness.

## Abbreviations

CHSCF: Comprehensive Health Seeking and Coping Framework
COVID-19: coronavirus disease 2019
HIV: human immunodeficiency virus
LGBTQ: lesbian, gay, bisexual, transgender, queer
STI: sexually transmitted infection
